# Book Reviews

**Published:** 1831-01

**Authors:** 


### CRITICAL ANALYSES.

Quæ laudanda forent, et quæ culpanda, vicissim

Illa, prius, cretâ; mox hæc, carbone, *notamus.*—Persius.

*An Introduction to the Natural System of Botany; or, a Systematic View of the Organisation, Natural Affinities, and Geographical Distribution, of the whole Vegetable Kingdom; together with the Uses of the most important Species in Medicine, the Arts, and Rural or Domestic Economy.* By John Lindley, f.r.s. l.s. g.s.; Member of the Imperial Academy Naturæ Curiosorum, of the Botanical Society of Ratisbon, of the Physiographical Society of Lund, of the Horticultural Society of Berlin; Honorary Member of the Lyceum of Natural History of New York, &c.; and Professor of Botany in the University of London. —8vo. pp. 374. London: Longman, 1830.

A work of no trifling character: it evinces much energy, labour, and research; and is highly creditable, both to the author and to the institution whose botanic chair he fills.

Having so lately, in our review of Lindley's "Synopsis" and "Outlines of the First Principles of Botany," expressed our decided opposition to the learned Professor's endeavour to render obsolete the Linnæan artificial system, and to supersede its use as an index by an awkward and degrading adaptation of the natural method; which, although admirable as the basis of a history of plants, becomes, as we then proved, a most ineffective guide, when used as a finder to those which are unknown; it would be a work of supererogation now again to traverse the same ground, merely because our author has not been converted by the arguments we then advanced, nor modelled his work entirely according to our advising. Moreover, as there is much in the details to applaud, we shall do little more than enter our protest against the principles of the general plan, upon which it would be useless further to descant; for,

"They who're convinced against their will,
Remain of the same opinion still."

Whether the observations on the Synopsis, published in our Journal,* led to many of the systematic alterations which occur in the present work, or whether these changes are the result of the author's more matured deliberation, we know not; but, in either case, the justice of our criticism

* London Med. and Phys. Journal, August 1830.

will equally be proved; on the one hand, by their spontaneous, on the other, by their compulsory, adoption. The dilemma likewise, which we then predicated, has occurred; for, although the definitions here are in general more correct, the diagnoses are more loose; the exceptions are also added, though covertly, under the head of "Anomalies," which anomalies subjoined to a large proportion of the orders form very numerous and important exceptions, being sometimes not only departures from, but absolutely contradictions to, the general rule; e.g. vide Order II. Umbelliferæ. The diagnosis says they are to be known by the possession of "an inferior didymous ovarium." The anomalies subsequently declare that "sometimes there are three carpella."

Order III. Ranunculaceæ. The diagnosis describes them as *polypetalous* dicotyledons, with *distinct* simple carpella, &c.; while the anomalies confess that, in some genera, "the carpella *cohere* more or less," and that, in Thalictrum, some species of Clematis, and some other genera, there are no petals.

Order IV. Papaveraceæ. "Diagnosis. *Polypetalous.*" "Anomalies. Bocconia has no petals," &c.

Order XII. Flacourtiaceæ. "Diagnosis. Polypetalous Dicotyledons," &c. "Anomalies. Ryania, Patrisia, Flacourtia, Roumea, and Stigmarota; that is to say, *more than half the order, have no petals.*"

Order XIII. Anonaceæ. "Diagnosis. Polypetalous . . . numerous, distinct, simple carpella. Anomalies. Monodora has a solitary carpellum. In Anona palustris, the ovaria are not distinct. Rollinia has the petals united. Stamens and carpella definite in Bocagea.

Orders XXV. XXVI. XXVII. XXIX. XXXII. XXXIII. XXXIV. XXXVIII. XXXIX. XLI. XLII. XLVI. XLVIII. L. LIII. LVI. and LVII. are in a similar predicament; and, when we find it written, as of Araliaceæ, Nymphæaceæ, &c. "*anomalies none,*" such orders often consist of very few genera, and sometimes, as in Nelumboneæ, of only *one.*

It is with regret that we notice, in the first page of the preface to this "Introduction to the Natural System of Botany," another anathema levelled at the Linnæan scheme, less violent, but not less virulent, than that which excited such general distaste in the "Synopsis." This seems to be the most obnoxious of all those "more popular classifications" which are described as being "well adapted, indeed, to captivate the superficial inquirer, but exercising so baneful an influence upon botany, as to have rendered it doubtful whether it even deserved a place among the sciences;" for afterwards "the celebrated sexual system" is stated to be the only one with which it is worth comparing the favorite natural mode. Upon these points we have formerly expressed ourselves somewhat fully; and our opinion is on record, that the natural and artificial schemes are, in truth, not hostile to each other; and that, so far from either being benefited, they would both be much injured by the abolition of the other.

This Linnæus, and Linnæan botanists, have willingly avowed, and the followers of Jussieu practically confess the same; for, by the addition of artificial clues to their natural schemes, they (according to their own showing) "render the natural system artificial:" and for what good end, when there is already a more perspicuous key, than any they ever have devised, familiar to the world at large? That it is more simple and perspicuous, we must still contend, and we believe that to this may be attributed its power of withstanding the reiterated attacks of its uncompromising assailants, more than to any "deeply-rooted prejudices with which, in a country like Great Britain, the natural system of botany has to contend." Against the proper application of the natural system, we know not that any prejudice exists: to its undue and exclusive use, a well-founded objection does certainly prevail, although even this is far less strenuously insisted on than the interests of botany demand: if any prejudice does exist, it still is, as it always has been, rather in favor of, than inimical to, the natural system, and the continual efforts to render this independent of that may be taken as a sufficient illustration. Of such prejudice let the present work suffice for proof: it affords an apposite, though not solitary, example.

The "uncertainty and difficulty" are, as stated by our author, the great objections to the use of the natural system as an index to any unknown plants; but, continues he, "it may be safely stated that it is not more uncertain than the celebrated sexual system of Linnæus." "By uncertain, I mean that the characters of the classes and orders of the natural system are not more subject to exceptions than those of the Linnæan; as, perhaps, may be proved from documents in the hands of every English reader." The proof then follows: it is a tabular conspectus of the exceptions occurring in several selected classes and orders as found in Smith's compendium, in which table the number of genera in each class and order is contrasted with the number of the same genera, which contain species that are exceptions to the general diagnostic rule. Thus we find that the class Monandria includes five British genera, three of which contain exceptional species; Triandria monogynia, nine genera, two having exceptional species; Tetrandria, twenty-one genera, with five exceptional species; Pentandria monogynia, forty-one genera, five having exceptional species, &c. But here it will be noted, in the first place, that these are selected sections, many classes and orders not being taken into the account: and, again, it cannot fail to be observed, that these departures in the Linnæan system from the diagnostic characters of the class and order are only exceptional *species*, frequently only one species out of an extensive genus; while the exceptions to the diagnoses of the natural orders, under the head of anomalies, include, along with anomalies properly so called, many departures from the characters of the classes and orders, not only of *species*, but, for the most part, of entire *genera*.

From such a partial view of the subject, little information can be drawn; the comparison should be based on other data. It is scarcely to be conceived that a whole genus must be condemned for one exceptional species; and when an author states that he "cannot forbear expressing his doubt whether any fourteen natural orders can be named, in which the proportion of exceptions is so considerable as in the fourteen selected from Smith's "Compendium," in which, out of 173 genera, forty-three contain exceptional species," he should have reflected on the fact that the very constitution of the Linnæan system precludes the possibility of exceptional *genera;* a great advantage, which the natural cannot boast; and that, were its *principle* strictly adhered to, exceptional species likewise would be all but unknown. In the artificial clue prefixed to Lindley's "Introduction," the same orders, in several instances, are enumerated twice: thus, Saxifrageœ are, in part, arranged in a section characterised by having the "calyx *adhering* more or less to the ovaria," and in part, in another section, which is distinguished from the foregoing by having the "calyx *distinct* from the ovarium." Vochyaceæ likewise are met with both in the *superior* and the *inferior* ovarian sections. Again, Penæaceæ, Apocyneæ, Epacrideæ, Plantagineæ, Loganiaceæ, Pedalineæ, and Ericeæ, appear in two different places; and this occurs with entire *orders:* why may not the same species be then enumerated occasionally twice? Moreover, the exceptional species are so arranged in the Linnæan system, that they are as easily distinguished as the normal ones: not so with the anomalous or exceptional genera of the natural scheme; there is in it no subsidiary clue for their reduction.

But we will accept the author's challenge on his own terms, though it is giving him greatly the "'vantage ground," and, in return, select a few natural orders, noting their exceptions from his own "Synopsis." The reference is given to the

"Synopsis," rather than to the "Introduction," because that work may, more justly than this, be compared to Smith's "Compendium;" both being confined to British plants.

The first order, Ranunculaceæ, contains fourteen genera, considerably above half of which are more or less anomalous in some, and several in all their species. Thus, in this Dichlamydeous polypetalous order, Thalictrum has *no petals* in any of its species; Clematis is apetalous in general; Anemone, Trollius, and Caltha, have sepals and petals so much alike, that they are described as "undistinguishable from each other;" and in Helleborus, Aquilegia, Delphinium, and Aconitum, their forms and characters are very doubtful. So that, in this first example, taken from the very first order, out of fourteen genera, nine are *practically*, if not altogether theoretically, exceptions.

We wish not to pursue this subject now: some further observations may be met with in the Number of this Journal we have before referred to, where it will be found that Caryophylleæ, Saxifrageæ, Ericeæ, Gentianeæ, Solaneæ, Scrophularineæ, Cupuliferæ, Coniferæ, Smilaceæ, &c. are more or less in the same predicament. Thus, Sanguisorbeæ, a section of Rosaceæ, contains but three *genera*, *all* of which are exceptions; and, in *all* their *species*, Hederaceæ contains but two genera, but *both* of which are exceptions; Pistiaceæ only a single genus, and that genus is, according to Lindley's definitions, exceptional in all its species. Halorageæ, Oleineæ, &c. might also be cited as further illustrations; but it would be useless; thus, Fraxinus is apetalous, and so is Glaux among the Primulaceæ: but of this ungracious task, enough. One observation more, and we have done.

The unsettled state of the natural system, a state inseparable from its rapid progress towards perfection, by which the knowledge of one day becomes ignorance the next, although the boast of this, as of every improving science, still forms no slight objection to its adoption as an initiatory study. On this point let our author speak; for he most truly says, the arrangement he has adopted "is not precisely that of any previous work; *nor, indeed, do any two botanists adopt exactly the same plan:*" and a still further proof might be given, not only by comparing the natural systems of any two botanists, but by comparing even Lindley with himself. "This is, however," (in his opinion,) "of no practical importance." We nevertheless cordially dissent from such a proposition; and, on the contrary, would hence infer, that some well-known standard of reference, as an index, (and we hold the Linnæan system as of no other value,) still is, and long will prove to be, a useful, nay an essential, helpmate to the higher philosophy of this enchanting study.

Of his own modification, our author modestly and truly declares, that "such a collection of orders cannot certainly be called the natural system of the vegetable kingdom, in the proper sense of these words; but it is what botanists take as a substitute for it, *until some fixed principle shall be discovered* upon which combinations can be formed, subordinate to the first great classes of Vasculares and Cellulares, of Exogenæ and Endogenæ." This is but the echo of our own ideas; for it is *only until* some fixed principle be discovered, or any other artificial sign, more commodious as an index than the sexual scheme, that we advocate the use of the Linnæan clue. Of the compatibility of such opposite perfections, we entertain, however, the strongest doubts.

We have said that the "Synopsis" and the "Introduction," works of the same author, and published only a few months apart, afford good illustrations of the unsettled state of the natural system: to them we refer the curious reader; for, with the same reckless indecision that Ranunculaceæ then formed the first order, it is now placed under Umbelliferæ, which, from then being the thirty-eighth order of the British, has now become the second of the entire Flora.

Numerous other similar promotions and degradations are to be noted: indeed, the list affords a good sample of a revolution, in which scarcely any order has been allowed to retain its ancient rank: the very bonds of the social compact appear to have been loosened; for the monopetalous plants now follow the Apetalæ and Achlamydeæ, which they formerly preceded; and, however natural the tribes Angiospermæ and Gymnospermæ may ultimately prove to be, they are any thing but convenient, in an analytical point of view, as the one contains 226 orders, and the other only 2: and, besides, the very first division of Vasculares, or flowering plants, and Cellulares, or flowerless plants, has much in it that is debateable; we think, objectionable. Not to waste time by enumerating all the exceptions, take one as an example: the 63d order, Cytineæ, is preceded by 62, and followed by 200 of confessed, we should say 204, orders of truly vascular plants; yet these plants contain *no spiral vessels*, the presence of which forms the grand distinction in the very first step of the analysis. Contrast the account given of the Vasculares by our author in this introduction, (taking either his artificial analysis of the orders, page xxxvii., or his essential characters of the same plants, page 1,) with the account given in the same work, pages 73, 74, of the 62d order Cytineæ.

"CLASS I. VASCULARES, OR FLOWERING PLANTS.

"Cotyledoneæ. *Juss. Gen.* p. 70. (1789.)—Embryonatæ, *Richard. Anal.* p. 50. (1808.) Vasculares, *Dec. Fl. Fr.* 1. 68. (1815); *Lindl. Synops.* p. 3. (1829.)—Phanerogamous *or* Phænogamous Plants *of authors.*

"*Essential character.* Substance of the plant composed of cellular tissue, woody fibre, ducts, and *spiral vessels.* Leaves composed of parenchyma, and of veins consisting of woody fibre and spiral vessels. Cuticle with stomata. Flowers consisting of floral envelopes, stamens, and pistilla. Seeds distinctly attached to a placenta, covered with a testa, and containing an embryo with one or more cotyledons; *germinating at two fixed points, the plumula and radicle.*

"The presence of flowers, of spiral vessels, and of cuticular stomata, will at all times distinguish these from Cellulares, or flowerless plants, in which ducts sometimes exist, but which never have spiral vessels. Vasculares approach Cellulares by Podostemeæ, some of which resemble Azolla in habit, by Fluviales, which are near Algæ, especially by Coniferæ and Cycadeæ, which are closely akin to Lycopodiaceæ and Filices, and also by Casuarina, which must, in any natural ordination, stand near Equisetaceæ. Besides the more obvious points of difference just adverted to, Vasculares differ from Cellulares in their embryo; not, however, in the number of the cotyledons, as is generally supposed in consequence of the common names of Dicotyledones, Monocotyledones, and Acotyledones, but in the germination of the seeds of the two former *always taking place from two fixed points,* and in the latter from no fixed point." (P. 1.)

"CLASS I. VASCULARES, OR FLOWERING PLANTS.

"Plants having distinct flowers and sexes.

"Sub-Class I. Exogenæ, or Dicotyledonous Plants.

"Leaves reticulated. Stem with wood, pith, bark, and medullary rays. Flowers with a quinary division. Cotyledons 2 or more, opposite." (P. xxxvii.)

"LXIII. CYTINEÆ.

"Cytineæ, *Adolphe Brongn. in Ann. des Sc. Nat.* 1. 29. (1824). Pistiaceæ *Agardh. Aphor. Bot.* p. 240. (1826).—Rhizantheæ, *Blume in Batav. Zeitung,* (1825); *Flora Javæ,* (1829).—Aristolochiæ, § Cytineæ, *Link Handb.* 1. 368. (1829.)

"*Diagnosis.* Apetalous leafless dicotyledons, with indefinite ovules, a 1-celled ovarium with parietal placentæ and indehiscent fruit.

"*Anomalies.* No spiral vessels exist in these plants.

"*Essential character.* Flowers diœcious, monœcious, or hermaphrodite. Calyx superior, with a limb divided into several divisions, which are imbricated in æstivation. Stamens cohering in a solid central column, from the apex of which arise some horned processes; anthers adnate, either bursting longitudinally and externally, or having their inside cellular, and discharging their pollen by orifices at the apex. Ovarium inferior, 1 or many-celled, with broad parietal placentæ, which are covered with an indefinite number of minute ovules. Fruit an inferior pulpy berry. Seeds extremely minute, (their nucleus consisting of a mass of grumous matter. *Blume.*) Parasitical brown on colourless plants, *without spiral* vessels. Stem simple, covered with a few leaves in the form of scales. Flowers in spikes or heads, or solitary." (P. 73.)

"But the most interesting circumstance of their organization is, that they exhibit in some degree the structure both of flowering and flowerless, or of vascular and cellular plants. Like flowering or vascular plants, they have a distinct floral envelope, and distinct sexual organs, not essentially, or in fact very, different from thoes of ordinary vegetables. Like flowerless or cellular plants, they are destitute of all trace of spiral vessels, and their seeds appear to be eomposed of a homogeneous mass of grumous matter, *in which no radicle or cotyledons, no ascending or descending extremity, no definite points of vegetation, can be distinguished.*" (P. 74.)

Can any diagnostic characters be more diametrically inconsistent with each other? It used to be taught, that every major proposition should include its minor; yet are the characters of this order included either in those of the sub-class Exogenæ, or in those of the class Vasculares, in which it is arranged? The great associations, both Exogenæ, or Dicotyledons, and Endogenæ, or Monocotyledons, are defined to have spiral vessels, and to germinate ALWAYS from *two fixed points;* while Cytineæ are stated to be "destitute of all trace of spiral vessels;" and, furthermore, it is added, "their seeds appear to be composed of a homogeneous mass of grumous matter, *in which no radicle or cotyledons, no ascending or descending extremity, no definite points of vegetation, can be distinguished.*"

We forbear citing more examples. The fault lies not in the system, but in its improper application: our own labours in the cause of the natural affinities of organic beings, will shield us from the charge of being inimical to it. Indeed, we think, we are its truest friends, when we attempt to restrain it to that province where its energies may be most advantageously employed.

The mode in which the particular orders are described, is excellent, so far as it goes; but while the synonymes of many authors are given, why have those of the natural orders of Linnæus been omitted? These should, at any rate, have been introduced, even if the artificial classes and orders could not be borne; although both, would have added, in our opinion, to the value of the work. The addition could have been easily made, and would have rendered the volume much more intelligible and useful: may we request them, in the next edition, to be introduced? Can any thing be more cumbrous, complex, and confused, than the artificial analysis prefixed to the supposed natural series of the orders? was it ever intended to be practically useful? was it ever expected that a student should find out, by its guidance, the names of unknown plants?

We have said that the mode in which the particular orders are described, is in general excellent: a great deal of very useful information is conveyed in few words, and in a very attractive guise. Let us justify our opinion, by quoting a few examples, taken almost indifferently from various sections of the book; and, after the perusal of which, we need scarcely add, that we recommend it strongly to all botanical students, whether disciples of the artificial or the natural schools; terms which we hope shortly to hear employed only in conjunction and amity with each other.

"III. Ranunculaceæ. The Crow-foot Tribe.

"Ranunculi, *Juss. Gen.* (1789.)—Ranunculaceæ, *Dec. Syst.* 1. 127. (1818); *Prodr.* 1.2. (1824): *Lindl. Synops.* p. 7. (1829.)

"*Diagnosis*. Polypetalous dicotyledons, with hypogynous stamens, anthers bursting by longitudinal slits, several distinct simple carpella, exstipulate leaves sheathing at their base, solid albumen, and seeds without arillus.

"*Anomalies*. In Garidella and Nigella the carpella cohere more or less. In Thalictrum, some species of Clematis, and some other genera, there are no petals. Pæonia has a persistent calyx.

"*Essential character*. Sepals 3-6, hypogynous, deciduous, generally imbricate in æstivation, occasionally valvate or duplicate. Petals 5-15, hypogynous, in one or more rows, distinct, sometimes deformed in correspondence with metamorphosis in the stamens. Stamens indefinite in number, hypogynous; anthers adnate, in the true genera turned outwards. Pistilla numerous, seated on a torus, 1-celled or united into a single many-celled pistillum; ovarium one or more seeded, the ovula adhering to the inner edge; style one to each ovarium, short, simple. Fruit either consisting of dry nuts or caryopsides, or baccate with one or more seeds, or follicular with one or two valves. Seeds albuminous; when solitary, either erect or pendulous. Embryo minute. Albumen corneous.—Herbs, or very rarely shrubs. Leaves alternate or opposite, generally divided, with the petiole dilated and forming a sheath half clasping the stem. Hairs, if any, simple. Inflorescence variable.

"*Affinities*. This is an order which has a strong affinity with many others, some of which are widely apart from each other. Its most immediate resemblance is with Dilleniaceæ, Magnoliaceæ, and their allies, to which it approaches in the position, number, and structure of its parts of fructification generally, differing however in an abundance of particulars; as from Dilleniaceæ, in the want of arillus, deciduous calyx, and whole habit; from Magnoliaceæ, in the want of stipulæ, and sensible qualities; from Papaveraceæ and Nymphæaceæ, in the distinct, not concrete, carpella, watery, not milky, fluids, acrid, not narcotic, properties. More distant analogy may be traced with Rosaceæ, with which they agree in their numerous carpella, the number of their floral divisions and indefinite stamens; but differ in those stamens being hypogynous instead of perigynous, in the presence of large albumen surrounding a minute embryo, want of stipulæ and acrid properties. With Umbelliferæ they accord in the last particular, and also in their sheathing leaves, habit, and abundant albumen, with a minute embryo; but those plants differ in their calyx being concrete with the ovarium, and in their stamens being invariably definite; no doubt, however, can be entertained, that in any really natural arrangement Ranunculaceæ and Umbelliferæ should be placed near each other. Another analogy has been indicated by botanists between this order and Alismaceæ, with which it agrees in its numerous ovaria, and in habit; but that order is monocotyledonous. A great peculiarity of Ranunculaceæ consists in the strong tendency exhibited by many of the genera to produce the sepals, petals, and stamens, in a state different from that of other plants; as, for example, in Delphinium, Aquilegia, and Aconitum, in which they are furnished with a spur, and in Ranunculus itself, which has a nectariferous gland at the base of the petals. An instance is described of the polypetalous regular corolla of Clematis viticella being changed into a monopetalous irregular one, like that of Labiatæ. *Nov. Act. Acad. N. C.* 14. p. 642. t. 37.

*Geography.* The largest proportion of this order is found in Europe, which contains more than 1-5th of the whole; North America possesses about 1-7th, India 1-25th, South America 1-17th; very few are found in Africa, except upon the shores of the Mediterranean: eighteen species have, according to Decandolle, been discovered in New Holland. They characterize a cold damp climate, and are, when met with in the Tropics, found inhabiting the sides and summits of lofty mountains; in the lowlands of hot countries they are almost unknown.

" *Properties.* Acridity, causticity, and poison, are the general characters of this suspicious order, which, however, contains species in which those qualities are so little developed as to be innoxious. The caustic principle is, according to Krapfen, as cited by Decandolle, of a very singular nature; it is so volatile that, in most cases, simple drying, infusion in water, or boiling, are sufficient to dissipate it; it is neither acid nor alkaline; it is increased by acids, sugar, honey, wine, spirit, &c. and is only effectually destroyed by water. The leaves of Knowltonia vesicatoria are used as vesicatories in Southern Africa. Ranunculus glacialis is a powerful sudorific; Aconitum Napellus and Cammarum are diuretic. The Hepatica,

Actæa racemosa, and Delphinium consolida, are regarded as simple astringents, *Dec.* The roots of several Hellebores are drastric purgatives; those of the perennial Adonises are, according to Pallas, emmenagogues; and those of several Aconitums, especially Napellus and Cammarum, are acrid in a high degree. *Ibid.* The root of the Aconitum of India, one of the substances called Bikh, or Bish, is a most virulent poison. *Trans. Med. and Phil. Soc. Calc.* 2, 407. Authors are, however, not well agreed what the precise plant is which produces this Bikh, although all agree in referring it to Ranunculaceæ. In India, it seems there are three principal kinds of Bish, varying from each other in their properties, but all belonging to a genus which Dr. Hamilton refers to Caltha. According to this author, the Bishma, or Bikhma, is a strong bitter, very powerful in the cure of fevers: the Bish, Bikh, or Kodoya Bikh, has a root possessing poisonous properties, of the most dreadful kind, whether taken into the stomach, or applied to wounds: the Nir Bishi, or Nirbikhi, has no deleterious properties, but is used in medicine. *Brewster*, 1, 250. For some important information on this Bikh, Vish, Visha, or Ativisha, which Dr. Wallich considers his Aconitum ferox, see *Plant. As. Rar.* vol. 1, p. 33, tab. 41. The root of Pæony is acrid and bitter, but is said to possess antispasmodic properties. Ranunculus flammula and sceleratus are powerful epispastics, and are used as such in the Hebrides, producing a blister in about an hour and a half. Their action is, however, too violent, and the blisters are difficult to heal, being apt to pass into irritable ulcers. *Ed. Ph. J.* 6, 156. Beggars use them for the purpose of forming artificial ulcers, and also the leaves of Clematis recta and flammula. From the seeds of Delphinium staphysagria, the chemical principle called Delphine was procured by MM. Lassaigne and Fenuelle; it exists in union with oxalic acid. *Ibid.* 3, 305. The root of Hydratis canadensis has a strong and somewhat narcotic smell, and is exceedingly bitter; it is used in North America as a tonic, under the name of Yellow root. *Barton*, 2, 21. The root of Coptis trifolia, or Gold-thread, is a pure and powerful bitter, devoid of any thing like astringency; it is a popular remedy in the United States for aphthous affections of the mouth in children. *Ibid.* 2, 100. The wood and bark of Xanthorhiza apiifolia are a very pure tonic bitter. The shrub contains both a gum and resin, each of which is intensely bitter. *Ibid.* 2, 205. The seeds of Nigella sativa were formerly employed instead of pepper; those of Delphinium Staphisagria are vermifugal and caustic; those of Aquilegia are simply tonic. *Dec.*

" M. Decandolle makes the following divisions in this order:

" I. True Ranunculaceæ.

" Anthers bursting outwardly.

" § 1. Clematideæ. *Dec. Syst.* 1, 131, (1818); *Prodr.* 1, 2, (1824.)

" Æstivation of the calyx valvate, or induplicate. Petals none, or plane. Carpella indehiscent, 1-seeded, terminated by a bearded tail (which is the indurated style). Seed pendulous. Leaves opposite.

" *Examples.* Clematis, Naravelia.

" § 2. Anemoneæ. *Dec. Syst.* 1, 168, (1818); *Prodr.* 1, 10, (1825.)

" Æstivation of calyx and corolla imbricated. Petals, none, or plane. Carpella 1-seeded, indehiscent, usually terminated by a tail or point. Seed pendulous. Leaves radical, or alternate.

" *Examples.* Anemone, Thalictrum.

" § 3. Ranunculeæ. *Dec. Syst.* 1, 228, (1818); *Prodr.* 1, 25, (1824.)

" Æstivation of calyx and corolla imbricated. Petals 2-lipped, or furnished with an anterior scale at the base. Carpella 1-seeded, dry, indehiscent. Seed erect. Leaves radical, or alternate.

" *Examples.* Ranunculus, Myosurus.

" § 4. Helleboreæ. *Dec. Syst.* 1, 306, (1818); *Prodr.* 1, 44, (1824.)

" Æstivation of calyx and corolla imbricated. Petals either none, or irregular, 2-lipped, and nectariferous. Calyx petaloid. Carpella capsular, dehiscent, many-seeded.

" *Examples.* Eranthis, Trollius, Aconitum.

" II. Spurious Ranunculaceæ.

" Anthers bursting inwardly.

" *Examples.* Actæa, Xanthorhiza, Pæonia. (P. 6.)

The theory of the formation of the fruit of the Pomegranate is ingenious, and is a favorable specimen of our author's phytological acumen.

" Punica is usually referred to this order; but the descriptions that have been published of it have been founded upon so imperfect a view of its structure, that I may be permitted to dwell upon it at some length, especially as I hope to show that it not only does not differ from the order essentially, but that it does not require to be distinguished from true Myrtaceæ, even as a section. A consideration of the real structure of this plant comes the more properly within the scope of the present publication, because the genus has been considered the type of a particular order (Granateæ) by Mr. Don, in which he is supported by the high authority of Decandolle and Von Martius. The fruit of the Pomegranate is described by Gaertner and Decandolle as being divided into two unequal divisions by a horizontal diaphragm, the upper half of which consists of from five to nine cells, and the lower of three; the cells of both being separated by membranous dissepiments; the placentæ of the upper half proceeding from the back to the centre, and of the lower irregularly from their bottom; and, by Mr. Don, as a fleshy receptacle formed by the tube of the calyx into a unilocular berry, filled with a spongy placenta, which is hollowed out into a number of irregular cells. In fact, if a Pomegranate is examined, it will be found to agree, more or less perfectly, with both these descriptions.

But it is clear that a fruit, as thus described, is at variance with all the known laws upon which compound fruits are formed. Nothing, however, is more common than that the primitive construction of fruits is obscured by the additions, or suppressions, or alterations, which the parts undergo during their progress to maturity. Hence it is always desirable to obtain a clear idea of the structure of the ovarium of all fruits which do not obviously agree with the ordinary laws of carpological composition. Now, a section of the ovarium of the Pomegranate, in various directions, if made about the time of the expansion of the flowers, before impregnation takes place, shows that it is, in fact, composed of two rows of carpella, of which three or four surround the axis, and are placed in the bottom of the tube of the calyx, and a number, varying from five to ten, surround these, and adhere to the upper part of the tube of the calyx. The placentæ of these carpella contract an irregular kind of adhesion with the back and front of their cells, and thus give the position ultimately acquired by the seeds, that anomalous appearance which it assumes in the ripe fruit. If this view of the structure of the Pomegranate be correct, its peculiarity consists in this, that in an order, the carpella of which occupy but a single row around the axis, it possesses carpella in two rows, the one above the other, in consequence of the contraction of the tube of the calyx, from which they arise.

"Now, there are many instances of a similar anomaly among genera of the same order, and they exist even among species of the same genus. Examples of the latter are, Nicotiana multivalvis and Nolana paradoxa, and of the former, Malope among Malvaceæ; polycarpous Ranunculaceæ, as compared with Nigella, and polycarpous Rosaceæ, as compared with Spiræa.

"In Prunus I have seen a monstrous flower producing a number of carpella around the central one, and also, in consequence of the situation, upon the calyx above it; and, finally, in the Revue Encyclopédique (43. 702.), a permanent variety of the apple is described, which is exactly to Pomaceæ what Punica is to Myrtaceæ. This plant has regularly fourteen styles and fourteen cells, arranged in two horizontal parallel planes, namely, five in the middle, and nine on the outside, smaller and nearer the top; a circumstance which is evidently to be explained by the presence of an outer series of carpella, and not upon the extravagant hypothesis of M. Tillette de Clermont, who fancies that it is due to the cohesion of three flowers. The anomaly of the structure of the fruit of Punica being thus explained, nothing remains to distinguish it from Myrtaceæ but its leaves without a marginal vein, its convolute cotyledons, and pulpy seeds. There are, however, distinct traces of dots in the leaves, and the union of the venæ arcuatæ, which gives the appearance of a marginal vein to Myrtaceæ, takes place, although less regularly, in Punica; the convolute cotyledons of Punica are only in Myrtaceæ what those of Chamæmeles are in Pomaceæ, a curious but unimportant exception to the general structure; and the solitary character of the pulpy coat of the seeds will hardly be deemed by itself sufficient to characterise Granateæ. The place of Punica in the order will be probably near Sonneratia." (P. 64.)

We regret the omission of any part of the following section, but it is too long to extract entire: we therefore select the most interesting and important passages.

"LXXVII. LEGUMINOSÆ. THE PEA TRIBE.

"Leguminosæ, *Juss. Gen.* 345. (1789); *Brown Diss.* (1822); *Dec. Prodr.* 2, 93. (1825); *Lindl. Synops.* 75. (1829.)

"*Diagnosis.* Polypetalous dicotyledons, with a terminal style, and solitary simple superior ovarium, perigynous definite stamens, exalbuminous seeds, peritropal ovula, leguminous fruit, and alternate stipulate leaves.

"*Anomalies.* The Detariums are apetalous and drupaceous. Ceratonia, Copaifera, and five or six other genera, are also apetalous. Some Mimoseæ are monopetalous; the latter section and Swartzieæ have usually also hypogynous stamens. Diphaca, and a species of Cæsalpinia, have regularly two ovaria. Ormosia has two stigmas. *Dec.* Sophora, Myrospermum, and some others, have no stipulæ. Some have opposite leaves." (P. 87.)

"*Properties.* This order is not only among the most extensive that are known, but also one of the most important to man, with reference to the objects either of ornament, of utility, or of nutriment, which it comprehends. When we reflect that the Cercis, which renders the gardens of Turkey resplendent with its myriads of purple flowers; the Acacia, not less valued for its airy foliage and elegant blossoms, than for its hard and durable wood; the Braziletto, Logwood, and Rosewoods, of commerce; the Laburnum; the classical Cytisus; the Furze and the Broom, both the pride of the otherwise dreary heaths of Europe; the Bean, the Pea, the Vetch, the Clover, the Trefoil, the Lucerne, all staple articles of culture by the farmer, are all species of Leguminosæ; and that the gums Arabic and Kino, and various precious medicinal drugs, not to mention Indigo, the most useful of all dyes, are products of other species; it will be perceived that it would be difficult to point out an order with greater claims upon the attention. It would be in vain to attempt to enumerate all its useful plants or products, in lieu of which I shall speak of the most remarkable, and of those which are least known.

"The beauty of Dr. Wallich's Amherstia nobilis, a large tree bearing pendulous racemes of deep scarlet flowers, is unequalled in the vegetable kingdom. The general character of the order is to be eminently wholesome; but there are some singular exceptions to this. The seeds of Lathyrus Aphaca are said to produce intense headach, if eaten abundantly; the seeds of the Laburnum are poisonous; they contain a principle called Cytisine. The root of a species of Minosa, called Spongia, is accounted a poison in Brazil. *Ed. P. J.* 14. 267. The leaves and branches of Tephrosia are used for intoxicating fish; the leaves of Ornithopus scorpioides are capable of being employed as vesicatories. The juice of Coronilla varia is poisonous. *Dec.* The powerful purgative effects of Senna are possessed also by other species, even by Colutea aborescens and Coronilla emerus. Cassia marilandica is found in North America a useful substitute for the Alexandrian Senna. *Barton.* 1. 143. The senna of the shops consists, according to M. Delile, of Cassia acutifolia, Cassia Senna, and Cynanchum Argel. He says the Cassia lanceolata of Arabia does not yield the Senna of commerce. The active principle of Senna is called Cathartine. It was discovered by MM. Lassaigne and Fenuelle. *Ed. P. J.* 7. 389. Purgative properties are also found in the pulp within the fruit of Cathartocarpus fistula and Ceratonia siliqua, of Mimosa fagifolia, and also of the Tamarind, the preserved pulp of which is so well known as a delicious confection. Malic acid exists in the Tamarind, mixed with tartaric and citric acids. *Turner*, 634. The same may be said of Inga fæculifera, or the Pois doux, of St. Domingo, that bears pods filled with a sweet pulp, which the natives use. *Hamilt. Prodr.* 62. The roots of the liquorice contain an abundance of a sweet subacrid mucilaginous juice, which is much esteemed as a pectoral; similar qualities are ascribed to Trifolium alpinum roots. The root of Abrus precatorius possesses exactly the properties of the liquorice root of the shops. *Ainslie*, 2. 79. In Java it is found demulcent. The seeds are considered by some as ophthalmic and cephalic, externally applied. The roots of Beans, Genistas, Ononis, Guilandina nuga and moringa, Anthyllis cretica, &c. are diuretic. *Dec.* Those of Dolichos tuberosus and bulbosus and Lathyrus tuberosus, are wholesome food. Some are reported to produce powerfully bitter and tonic effects. Various species of Geoffræa, the bark of Æschinomene grandiflora and of Cæsalpinia bonduccella, are of this class. The kernels of Guilandina bonduccella are very bitter, and are supposed by the native doctors of India to possess powerful tonic virtues. When pounded small, and mixed with castor oil, they form a valuable external application in incipient hydrocele. *Ainslie*, 2. 136. The leaves are a valuable discutient, fried with a little castor oil, in cases of hernia humoralis. *Ibid.* The bark of Acacia arabica is considered in India a powerful tonic: a decoction of its pods is used as a substitute for that of the seeds of Mimosa saponaria for washing. *Ibid.* 2. 142. The root of Hedysarum sennoides is accounted in India tonic and stimulant. *Ibid.* 2. 53. These powers are probably connected with the astringent and tanning properties of several others. Some of the Algarobas or Prosopises of the western part of South America bear fruit, the pericarp of which consists almost wholly of tannin. The bark of some of the species of Acacia abound to such a degree in tanning principles, as to have become objects of commercial importance. In 1824, some tons of the extract of Acacia bark were imported from New South Wales, for the use of tanners. *Ed. P. J.* 11. 266. The pods of Cassia Sabak and Acacia nilotica are used in Nubia for tanning. *Delile Cent.* 10.

The valuable astringent substance, called Catechu, or Terra Japonica, is procured by boiling and evaporating the brown heart-wood of Acacia Catechu, or Kair tree: it is obtained by simply boiling the chips in water until the inspissated juice has acquired a proper consistency; the liquor is then strained, and soon coagulates into a mass. *Brewster*, 5. 349. Gum Kino is the produce of Pterocarpus erinacea, *R. Br.*, Gum Dragon and Sandalwood of Pterocarpus Draco and Santalinus, Gum Lac of Erythrina monosperma, Gum Anime of Hymenæa Courbaril, *Dec.*, Gum Arabic is yielded by Acacia senegalensis and some others, Gum Tragacanth by Astragalus creticus and similar species. According to Mr. Don (*Prodr.* no. 247.), the Manna of Arabia is produced by several species of Hedysarum, related to H. Alhagi. The Dalbergia monetaria of Linnæus yields a resin very similar to Dragon's Blood. *Ainslie*, 1. 115. A similar juice is yielded by Butea frondosa and superba. *Dec.* Among the woods of trees of this order, the most important is that of the Locust Tree, Robinia pseudacacia, which is a light bright yellow, hard and durable, but brittle. The Brazil wood of commerce is obtained from Cæsalpinia Braziliensis. The fine Jacaranda, or Rosewood of commerce, so called because when fresh it has a faint but agreeable smell of roses, is produced by a species of Minosa in the forests of Brazil. *Pr. Max. Trav.* 69. Among dyes are Indigo, produced by all Indigoferas and some Galegas, Logwood, the wood of Hæmatoxylon campeachianum, and the red dye yielded by several Cæsalpinias. The colouring matter of logwood is a peculiar principle, called Hæmatin. The wood of Pterocarpus santalinus yields a deep red colouring matter; it is known in commerce under the name of Saunders wood. *Ainslie.* 1. 386. All the species of the genus Copaifera, and sixteen are known, yield the balsam of copaiva; but it is not in all of them of equal quality. C. multijuga is said, by Von Martius, to afford the greatest abundance. *Hayne in Linnæa*, 1826. 418. The balsam is known in Venezuela under the name of Tacamahaca. *Dec. Podr.* 2. 508. Myroxylon peruiferum, the Quinquino of Peru, produces a fragrant resin, in much use both for burning as a perfume and for medicinal purposes, called the balsam of Tolu. *Lambert's Illustr.* 95. Both it and the balsam of Peru are also yielded, according to Ach. Richard, by M. toluiferum. *Ann. des Sc.* 2. 172. The root of Clitoria ternatea is emetic. *Ainslie*, 2. 140. The seed of Psoralea corylifolia is considered by the native practitioners of India stomachic and deobstruent. *Ibid.* 141. According to Dr. Horsfield, the Acacia scandens of Java is classed among the emetics. *Ibid.* 2. 108. The roots and herbage of Baptisia tinctoria have been found to possess antiseptic and subastringent properties. They have also a cathartic and emetic effect. *Barton*, 2. 57. The seeds of Cassia auriculata are considered by the Indian doctors as refrigerant and attenuant. *Ainslie*, 2. 32. The leaves of Coronilla picta are highly esteemed among the Hindoos, on account of the virtues they are said to possess in hastening suppuration, when applied in the form of a poultice; that is, simply made warm, and moistened with a little castor oil. *Ibid.* 2. 64. The seeds of Parkia africana are roasted as we roast coffee, then bruised, and allowed to ferment in water. When they begin to become putrid, they are well washed and pounded; the powder is made into cakes, somewhat in the fashion of our chocolate; they form an excellent sauce for all kinds of meat. The farinaceous matter surrounding the seeds forms a pleasant drink, and they also make it into a sweetmeat. *Brown in Denham*, 29. The irritating effects of the hairs which clothe the pods of Dolichus pruriens, or cowhage, are well known. A strong infusion of the root of the same plant, sweetened with honey, is used by the native practitioners of India in cases of cholera morbus. *Ainslie*, 1. 93. The native practitioners in India prescribe the dried buds and young flowers of Bauhinia tomentosa in certain dysenteric affections. *Ibid.* 2. 48. A decoction of the bitter root of Galega purpurea (Tephrosia) is prescribed by the Indian doctors in cases of dyspepsia, lientery, and tympanitis. *Ibid.* 2. 49. The powdered leaf of Indigofera anil is used in hepatitis. *Ibid.* 1. 179. The volatile oil of the Commarouma odorata, or Tonka bean, has been ascertained to be a peculiar principle called Coumarin. It was mistaken by M. Vogel for Benzoic acid. *Turner*, 660. It may be found in a crystallised state between the skin and the kernel, and exists abundantly in the flowers of Melilotus officinalis. *Ed. P. J.* 3. 407. It has been found that a peculiar acid, called Carbazotic, is formed by the action of nitric acid upon Indigo. *Turner*, 641. Sulphur exists in combination with different bases in peas and beans. *Ed. P. J.* 14. 172. The leaves of the Phaseolus trilobus (called Sem, or Simbi,) are considered by Indian practitioners cooling, sedative, antibilious, and tonic, and useful as an application to weak eyes. *Trans. M. and P. Soc. Cal.* 2. 406." (P. 90.)

The general principle that similar qualities are to be found pervading all the plants of the same natural order, has been so frequently insisted on, that it has almost degenerated into a prejudice: the exceptions are, however, too frequent to be disregarded. We are glad to see them now admitted; for we know that, when the noxious properties of one of the papilionaceous plants was mentioned to a very learned botanist, he at once crushed the supposition, by declaring it to be "*impossible; to be contrary to nature.*" It is nevertheless true, notwithstanding the impossibility, that cattle are injured by feeding on the Cytisus laburnum: a case in point has also been recorded by Mr. North,* in which a child was seen by him, labouring under the ordinary symptoms of poisoning, who had eaten some flowers of laburnum, which it had gathered from a beaupot. This important circumstance might

* London Med. and Phys. Journal, July 1829, p. 86. Dr. A. T. Thomson published some "Remarks on the Poisonous Properties of the Laburnum," in our Number for August 1829, p. 93. Editor.

well have been introduced; for the text would lead to the supposition that the seeds alone are poisonous. It is likewise to be found mentioned in the Dict. des Sciences Medicales.*

We did once purpose to have reviewed LOUDON's "Hortus Britannicus" in the present Number; but we prefer postponing a notice of it, until we can lay before our readers an account of another work of the same indefatigable bookwright, viz. his "Encyclopedia of Plants."

---

*Cases illustrative of the Efficacy of various Medicines administered by Inhalation, in Pulmonary Consumption; in certain Morbid States of the Trachea and Bronchial Tubes, attended with distressing Cough; and in Asthma.* By Sir CHARLES SCUDAMORE, M.D. F.R.S.; Honorary Member of Trinity College, Dublin, of the Medico-Chirurgical Society of Edinburgh, and of the Medical Society of Paris; Physician in ordinary to his Royal Highness the Prince Leopold of Saxe Coburg; Physician Extraordinary to his Excellency the Lord Lieutenant of Ireland. 8vo. pp. 118. Longman and Co., London, 1830.

IF we are not much mistaken, the fate of this work will, in one respect, be rather singular. There can be but little difference of opinion as to the rank which ought to be assigned to it in the estimation of the profession. We would willingly, indeed, reject the impression which its title, style, and general contents, force so strongly upon the mind; but it is impossible. Mr. St. John Long has stumbled in the midst of his prosperous career: his inhalations have been blown upon; his counter-irritant has been tried both by law and physic, and it has proved to be a little too powerful. Upon the professional exit of this lamentably celebrated character, Sir CHARLES SCUDAMORE steps in with new *vapours* and a very safe and agreeable external medicament. It must be confessed that the moment Sir Charles has chosen for his *entrée* in the rubbing and inhaling line is very judicious. From all we have learnt of the public opinion in reference to St. John Long's practice, we believe that the principles of his treatment are still very much admired, but that his practical weapons are deemed somewhat too rough. Now, Sir Charles Scudamore wields more gentle arms, but of the "self-same nature;" and, as far as we can discover from the present work, the principles which induce him to employ them differ but little from those of Mr. Long. It is more than probable, therefore, that many who are yet half inclined, but still afraid, to give Mr. Long another

* Dict. des Sc. Med. t. xlv. p. 187. ED.

trial, may prefer the safer and yet very similar treatment which it is the object of this work especially to recommend. Far be it from us positively to assert, that Sir Charles Scudamore has ever for a moment thought of the golden harvest he might reap, by occupying the void which has been produced by the melancholy decline of Mr. Long's reputation. He may have once more been induced to throw down the gauntlet as an author, from the purest and most philanthropic of motives; but whoever peruses these "cases illustrative" must feel with us that appearances are very strongly in favor of the supposition, that he is angling for the stragglers from Mr. Long's now deserted camp. But, notwithstanding this is the feeling the work will probably create, we must neither leave ungratified the curiosity of our readers, nor commit an act of injustice upon Sir Charles, by altogether passing over the evidence he brings forward in favor of the plan he proposes.

We deem it proper to give a general sketch of the contents of the work.

Having failed in the trial of all the means with which he was acquainted for the cure of pulmonary and bronchial disease, Sir C. was induced, about two years ago, to have recourse "to that new and extraordinary medicine termed iodine." Of this remedy he employed a preparation "miscible with hot water, so as to remain in permanent solution, using for the purpose of inhalation a glass apparatus, well fitted in its construction for the exhibition of the remedy in the form of vapour. The first cases in which it was tried were too desperate to admit of cure; but the palliative powers of the treatment were shown, and also its entire and perfect safety to the patient. Extensive experience, Sir Charles assures us, has satisfactorily proved to him the great advantage of using various medicines by the mode of inhalation; iodine more especially.

The first case detailed was one of pththisis pulmonalis in the last stage; "the disorganization of the lungs evidently too extensive to allow any probability of cure, and the treatment adopted with the hope of mitigating the symptoms." The patient was afflicted with the ordinary symptoms of pulmonary consumption, and he was treated in the following manner:

"I prescribed a weak solution of iodine, with the addition of some saturated tincture of conium, mixed with water of 120 degrees of heat, to be inhaled for fifteen or twenty minutes, three times a day. I directed him to take a minim of a solution of acetate of morphia, containing a grain of the acetate in six minims, in a simple saline draught at bedtime, and to repeat this dose in an hour or two, if necessary; to regulate the bowels by simple means; to wash the chest and upper part of the back with a mixture consisting of two parts of water, one of eau de Cologne, and one of vinegar; dipping a towel in the lotion for that purpose." (P. 6.)

"On first using the inhalation, he experienccd slight giddiness for a few minutes, and some sense of soreness, with dryness in the tongue and throat; but the patient rather mentioned these sensations on being interrogated, than complained of them; and they did not continue. He soon found that it afforded him great relief, the power of expectorating being remarkably facilitated; the cough also very much abating; the respiration becoming comfortable; and the chest materially freed from oppression. In all respects, he improved in a surprising manner. At the end of a fortnight, the pulse ranged below 100; the looks and the strength were improved; and both he and his friends, flåttered by this rapid amendment, anticipated an eventual recovery of health." (P. 7.)

From exposure to cold, the cough again became exceedingly harassing, and "flying pains" in the chest came on. To relieve these symptoms, "leeches and a blister were applied, and the inhalation was changed for a mixture of conium with hydrocyanic acid." The cough was thus relieved, and at the end of a week the use of the iodine mixture was resumed. The patient often declared that he should "be suffocated with the phlegm," had he not been enabled to get rid of it by means of inhaling. In two months from the date of Sir C.'s first visit, the disease terminated in the death of the patient. Here, indeed, the only object was to put to the test the powers of inhalation to mitigate the symptoms in an incurable case. In the second case, also, disease of the chest, complicated with tubercles and ulceration in the intestines, had proceeded too far to admit of more than alleviation. An inhalation of iodine with conium, the sixth of a grain of acetate of morphia at night, and medicine in the day calculated to allay intestinal irritation, were prescribed, and the chest was washed with the lotion described in a previous extract. As nutritious diet was allowed as the weak digestive powers would allow.

"Extremely debilitated as this patient was, he could use the inhaler without difficulty; thus affording a proof of the convenience of this simple apparatus. The relief which was obtained from this process, in the course of a few days, was most remarkable, and such as to exceed my utmost expectations. The patient's description of the effects of inhaling was, that it abated the cough remarkably, and rendered the expectoration, which before had been much suppressed, easy and free; from which change ensued a comfortable state of chest, with a great improvement in the breathing. He observed, that he felt the inhalation very sensibly traverse the chest, causing an agreeable sense of warmth." (P. 15.)

By means of the acetate of morphia, the nights were passed in comfort. On former occasions, when opiates had been given, they disagreed so exceedingly, that the patient declared "the remedy was worse than the disease." The inhalation was regularly used "almost up to the period of his death; and he always described in strong terms the relief which it gave to the chest."

Sir Charles now proceeds "to the more agreeable task of relating cases serving to exhibit in favorable colours the principles of treatment which it is the object of these pages to recommend."

Case III. "Hæmoptysis succeeded by ulceration; hectic fever, well marked; from all concurrent symptoms, the existence of phthisis pulmonalis established; the curative powers of iodine inhalation strongly displayed." The following is the detail of this case.

"A female, aged thirty-four, of delicate form, with rather narrow, yet not an ill-formed chest, of fair complexion, with dark eyes and white teeth, the mother of several children, having been much debilitated by three miscarriages within the last two years, and suffering from a severe cough, consulted me in February of the present year. In the history of her case, she related that, four years ago, she first contracted a violent catarrhal cough, which had since continued always troublesome, with the exception of an intermission in the summer months; that in January she had coughed up blood to the amount of a teacupful; and from that time had been affected with constant cough, pains of the chest, with quickened and difficult respiration, frequent palpitation of the heart, inability to lie on the right side, and one very distinct paroxysm of hectic fever in the middle of the day, and a slighter one in the evening. There were copious night sweats; she was much wasted in flesh; the catamenia had been suspended two months; the pulse was 120; the animal heat 99°; the expectoration was in quantity about four ounces in the twenty-four hours, of a general puriform appearance, and gave a ring of colours in the optical experiment: the digestive functions were not much disturbed; but the urine deposited much lateritious sediment.

The following indications appeared from the stethoscope and percussion. The voice was brought distinctly under the tube at the apex of the right lung, and there was obscure gargouillement at that part. The sound was dull at the upper part of the right lung, and very remarkably so on percussing the clavicle. The left lung was comparatively in a healthy state.

I prescribed a weak solution of iodine for the inhalation; internally, from one to two minims of the solution of acetate of morphia; and the following draught before rising in the morning: ℞. Magn. Sulphat. ʒi.; Infus. Rosæ ʒxii.; Acidi Hydrocyan. ♏i.; Syrupi

Tolutan. ʒi. M. fiat haustus. The chest all round was washed, night and morning, with the compound vinegar lotion.

"The diet was limited to boiled fish, vegetables, and farinaceous puddings. At the end of a few days, she found herself improved, and particularly as to the greater facility of expectorating, more ease of chest, and better respiration. The cough, however, still being very irritable, I added conium to the inhalation.

"The mitigation of the symptoms was now very obvious, and at the end of a fortnight the amendment was great: but about this period she took cold, and suffered severely, for twenty-four hours, from disorder of the bowels, and from spasms, which appeared to proceed from uterine irritation. The cough became more irritable; but otherwise the pulmonary symptoms were not aggravated. I changed the inhaling mixture for one consisting of conium and prussic acid. This indisposition soon yielded to treatment, and the iodine inhalation, with conium, was resumed, and with an increased proportion of iodine. At the end of a month, her appearance was remarkably improved, and all the symptoms were relieved. The pulse was reduced to 80, the animal heat to 95°; the respiration appeared unembarrassed; the cough was comparatively slight; the sputa small in quantity, and much improved in character; there was no longer hectic fever, and the night sweats were much lessened. She had gained flesh, and some improvement of strength; yet she still complained of great debility.

"She had been most attentive in the use of the inhalation three times a day, and extolled it as the source of her improvement. For the last week she had discontinued the morphia at night, and took no other medicine than the mild aperient draught occasionally. The most urgent symptoms being subdued, I now directed my attention to the improvement of the strength. I prescribed the following draught: ℞. Acidi Hydrocyan. ♏i.; Decoct. Cinchon. ℥i.; Mist. Amygd. ℥ss.; Aquæ Menth. virid. ʒij. M. fiat haustus bis die sumendus.

"She was desired to use the inhalation only twice in the day. She took mild animal food each other day, and at dinner two ounces of old port in a tumbler of cold water. She continued the use of the vinegar lotion. She took carriage exercise when the weather was favorable, and walked out occasionally.

"In another fortnight I prescribed a saline bark draught, omitting the hydrocyanic acid, and allowed her to take meat or poultry every day. She continued to amend regularly. The catamenia returned. Three months having elapsed, she had recovered so completely that no further treatment appeared to be necessary. For the last week she had inhaled only once a day. She improved in flesh, and was so much stronger, that she declared herself better in health altogether than she had been for six or seven years.

"This lady having removed to a distant part of the country, I have no opportunity of ascertaining the present state of her chest by auscultation; but I have the satisfaction of hearing that she continues perfectly well." (P. 20.)

The fourth case related was one of "bronchitis attended with high irritation. The existence of tubercles questionable. The utility of inhalation sufficiently well shown, as materially assisting the removal of the symptoms." In conjunction with other means, "an inhaling mixture, composed of conium, hydrocyanic acid, and an alcoholic tincture of ipecacuanha," was used. The urgent symptoms being speedily relieved, Sir C. deemed it expedient to direct the iodine inhalation with conium, which was employed for twenty minutes three times a day. "At the end of a week, the proportion of the iodine was increased, and the conium was omitted;" as, from the diminution of all the symptoms, the narcotic ingredient was no longer necessary. To tell us that "the proportion of the iodine was increased," is not to much purpose, as we are left totally in the dark as to the quantity originally employed. As Sir Charles Scudamore, however, makes an effort to defend this discreet reserve at the end of the book, we shall defer any particular comment upon it until we arrive at the reasons he ventures to give in justification of his secrecy. The subsequent progress of this case is said to have been very satisfactory, and in the course of a few weeks the patient's health was well established.

In Case v. the patient had suffered some years from chronic cough, "apparently depending on tuberculous irritation." A cure was effected by the inhalation of iodine and hemlock; the chest was also washed with the compound vinegar lotion, rendered slightly tepid; and, when the surface was dried, the flesh brush was used as long as it could be borne without fatigue.

In this case Sir C. deems it reasonable to believe that tubercles existed, and "certainly, from no means that had ever before been tried, did any benefit arise comparable with that produced by inhalation."

In the sixth case, the symptoms appeared to indicate the existence of tubercles in each lung: the iodine inhalation, the lotion to the chest, and other means, were employed, and the patient recovered.

We perfectly agree with the author, that "it should be held as an axiom that general bloodletting is to be practised on consumptive patients with the utmost circumspection." We adverted to this important practical point in our last Number, in our review of Mr. Rennie's book.

Although Sir Charles places great dependence on the use of inhalations, he considers it, in most instances, "useful or necessary" to call the power of internal medicines to his aid.

"During the state of hectic irritation, I usually prescribe very small doses of the hydrocyanic acid, as from one to three minims, in combination with a saline nitre draught, or cooling saline aperient, as mentioned in this case; but I strongly object to the administration of considerable doses of this powerful agent. My purpose, with this medicine, as an auxiliary, is sufficiently answered by small doses, and which can be administered without apprehension of disagreeable consequences.

"I think it a valuable sedative, but do not attach any other importance to its properties; and I believe that the warmest advocates of this medicine, as a curative remedy in phthisis, have found themselves quite disappointed in its effects." (P. 50.)

The following remark merits attention, as it may guard the practitioner from forming too hasty a prognosis, merely from the appearances of the expectoration.

"The leading point which engages the mind of the practitioner, is the discrimination between pus and mucus. Undoubtedly the indications afforded by the sputa are very instructive, and it is of great importance that we should well understand the nature of their numerous varieties; but we should be careful not to form a precipitate prognosis from this kind of evidence, however clearly it may be understood. A patient may be in a state of hazard from tubercles, whose expectoration is entirely of a mucous nature; and, as in the case which I have just related, may recover, although the expectoration is unequivocally purulent." (P. 51.)

In inflammation of the urethra, and in the conjunctive membrane of the eye, proofs are afforded that the altered secretion may be entirely puriform in its nature, although there is no breach of surface. The difficulty of discriminating pus from mucus, is acknowledged. The expectorated matter may float on the surface of water, and yet contain pus; "for it is always more or less blended with mucus, and may be kept for a while from sinking by the bubbles of air which are mixed with the bronchial secretion." Sir Charles has bestowed much consideration upon this subject, and he prefers the optical experiment described and recommended by the late Dr. YOUNG.

"I employ two circular pieces of well-selected plate glass, interposing a small portion of the matter to be examined between them, and hold the glasses, placed closely and evenly together, before a wax taper; for, the clearer the light, the more distinct is the effect produced. If the sputa contain pus, circles of coloured light will appear, which, when well marked, are red and green alternately; but, if it be altogether a mucous secretion, no such colours are produced. Pure pus the more strikingly displays this resemblance of a small rainbow,* and the colours of the ring are well marked according to the proportion of true pus in the mass of matter. So far, then, we have a simple and ready mode of judging of the nature of the expectoration in pulmonary disease." (P. 54.)

The following mode of applying cantharides as a counter-irritant, is new to us, and may, we should think, be useful in some cases.

"I have used, with advantage, as a convenient counter-irritant, a saturated infusion of cantharides in strong acetic acid. It is a very manageable remedy, and in many ways highly convenient: if applied diluted, it will act as a rubefacient; if in its state of concentration, it will vesicate in a short time: it may be applied, by means of a camel's-hair brush, to the smallest extent of surface, and in any situation; and it is less formidable treatment, in appearance, than the ordinary blister." (P. 56.)

As a general principle of treatment in pulmonic diseases, the author is desirous of avoiding every kind of painful counter-irritant.

Ten other cases are related, for the same purpose as those to which we have referred, to show the benefit that may be expected in pulmonic diseases, certain affections of the larynx and bronchia, and in some cases of asthma, by the use of the iodine inhalation, either alone or in combination with conium, and other agents.

Sir Charles terminates his work with some "general observations and conclusions." He states, that some of the medicines he has recommended for inhalation are agents of much delicacy and power; but his conviction of their most perfect safety, when employed in this manner, has not been shaken by a single untoward instance.

"Their administration requires careful attention and management. The composition of an inhaling mixture, and the doses to be used, are to be adapted to the particular case, and changed according to its varying circumstances, in the same manner as we find it necessary and proper to alter and accommodate our treatment with internal medicines.

"For this reason, therefore, and from an apprehension that patients themselves might be tempted to undertake the treatment of their own cases, with the great risk of receiving injury instead of benefit, I have avoided the introduction of formulæ of the remedies for inhaling, although I have, on every occasion, mentioned them in a general manner." (P. 102.)

If this "reason" were admitted as a satisfactory apology

* "The explanation is simply this: that pus, containing globules, causes a refraction and reflection of the rays of light; while mucus is free from globules."

for the secrecy the author has chosen to maintain, every professional writer would be justified in keeping to, and *for*, himself any practical improvement his experience might lead him to place confidence in. It is evident that Sir Charles Scudamore feels conscious he leaves himself open to censure for not being more unreserved in the description of his plan of treatment; and it must be confessed he makes but a feeble attempt to escape from it. A few remarks are added, for the information of the professional reader, as to the mode of using the different inhalations recommended in the work. Of all these agents, the iodine is the most active, and that in which confidence is placed as the curative remedy in phthisis pulmonalis. What quantity of the iodine we are to employ in each inhalation, Sir Charles Scudamore declines to tell us, for the "reason" before mentioned.

If the public peruse this work, they must perceive that Sir Charles Scudamore alone is in possession of the "precise and graduated doses of the iodine." If the profession honour it by their perusal, and are satisfied by the evidence adduced of the efficacy of the treatment proposed, they must also refer to Sir Charles Scudamore for more definite instructions, before they can give it a trial. Whether these inhalations will be freely taken in by the public, is yet to be determined. If they should have a *long* run, Sir Charles must be the only gainer, unless he should be "whipped by reproof" into an explicit explanation of the precise mode of using the medicines he recommends. As far as we know, this is the first instance in which a regular and respectable physician has, from *any* motive, mystified the management of a peculiar remedy: for the honour of a liberal profession, we trust it will be the last.

---

*A Practical Treatise on the Diseases of the Eye.* By Wm. Mackenzie, Lecturer on the Eye in the University of Glasgow, &c.—8vo. pp. 876. London: Longman and Co. 1830.

To practitioners in general, such a work as the present must be peculiarly acceptable and useful; for it can but rarely happen that the ordinary routine of practice can furnish sufficient examples of diseases of the eye, to enable us to obtain, from our personal experience, either an adequate knowledge of their pathology or treatment. We must look to those, for instruction upon these important subjects, who have extensive opportunities of seeing and treating such diseases, and who have also the ability of turning to a profitable account the advantages of their experience. As surgeon to a large ophthalmic institution, the author of this work enjoys the most desirable field for observation, and he has shown, by various valuable contributions to ophthalmic surgery, how earnestly and effectually he cultivates it.

We shall offer no formal apology for not entering into a regular analysis of a work consisting of nearly a thousand thickly printed pages. As a specimen of the general manner in which the various subjects are discussed, we shall select one or two chapters upon particular diseases of the eye; but we must first premise, that *every* malady affecting this organ is carefully described, and that the author has added much to his personal information, by a very laborious and erudite examination of most English and foreign authorities that are worthy the honour of being referred to. We observe, with some surprise, that the name of GUTHRIE is but seldom mentioned. Mr. MACKENZIE would have done well to quote that gentleman's authority and experience more frequently upon various subjects connected with ophthalmic surgery. To give one out of many examples,—upon the subject of the effects arising from the Argenti Nitras. and Murias Hydrarg. ointments in some diseases of the eye, it would naturally have been expected that Mr. Guthrie's repeated experience of the utility of these ointments would have been taken notice of. Mr. G. has published, in our own and other journals, so many interesting cases illustrative of the advantages of these applications, that they can scarcely have been overlooked. Notwithstanding the note which Mr. Mackenzie has prefixed to his work, which in a great degree modifies the positive exclusion of the Arg. Nitr. ointment, which he advises at p. 327, it is very evident that he is yet but imperfectly acquainted with the proper strength or action of the remedy.* For further instruction upon these points, we may refer to our Number for September 1828, p. 193, in which Mr. Guthrie details many instances in which it was successfully employed, and also comments upon the different effects produced, according to the length of time the ointment had been made before it was used.

Mr. Mackenzie presents us with so many temptations to extract from his work, that we find it somewhat difficult to make a selection. The following observations upon double vision, from want of correspondence in the action of the muscles of the eyeball, are interesting.

* Mr. Mackenzie "has been induced" to try a salve, composed of five grains of nitrate of silver to an ounce of lard. Mr. Guthrie uses an ointment with from two to ten grains of the nitrate of silver to one drachm of simple cerate. (London Med. and Phys. Journal, September 1828.)—ED.

"In strabismus, there is a want of correspondence in the actions of the muscles of the eyeball, and at the commencement of the complaint, there is double vision; but it would appear, that double vision occasionally occurs with so very slight a degree of distortion of the eyes, as scarcely to be observable. The double vision to which I refer, takes its origin, at least in some cases, from over-exertion of the eyes, and is an affection of the muscles of the eye-ball. It is of importance to be aware of the existence of cases of this kind, lest we should confound them with those in which double vision is owing to an affection of the brain, or of the optic nerve.

"Sir Everard Home, who first pointed out the practical importance of this distinction, has related two cases as ilusltrative of the symptoms and treatment of the subject of this section. The cases are interesting in several respects, although it must be confessed that there is no very conclusive evidence to prove that the symptoms were dependent merely on an affection of the muscles of the eyeball, and not on the state of the brain.

"The first case which led him to pay attention to the subject, was that of a lieutenant-colonel of engineers, who was in perfect health, shooting moor-game upon his own estate in Scotland. He was very much surprised, towards the evening of a fatiguing day's sport, to find all at once that every thing appeared double; his gun, his horse, and the road, were all double. This appearance distressed him exceedingly, and he became alarmed lest he should not find his way home: in this, however, he succeeded, by giving the reins to his horse. After a night's rest the double vision was very much gone off; and in two or three days he went again to the moors, when his complaint returned in a more violent degree. He went to Edinburgh for the benefit of medical advice. The disease was referred to the eye itself, and treated accordingly; the head was shaved, blistered, and bled with leeches. He was put under a course of mercury, and kept upon a very spare diet. This plan was found to aggravate the symptoms; he therefore, after giving it a sufficient trial, returned home in despair, and shut himself up in his own house. He gradually left off all medicine, and lived as usual. His sight was during the whole time perfectly clear, and at the same time near objects appeared single; at three yards they became double, and by increasing the distance, they separated farther from each other. When he looked at an object, it was perceived by a by-stander, that the two eyes were not equally directed to it. The complaint was most violent in the morning, and became better after dinner, when he had drank a few glasses of wine. It continued for nearly a twelvemonth, and gradually went off.

"Some time after the recovery of this gentleman, a house-painter, who had worked a good deal in white lead, was admitted a patient into St. George's Hospital, on account of a fever, attended with violent headach. Upon recovering from the fever, he was very much distressed at seeing every thing double; and as the fever was entirely gone, he was put under Sir Everard's care for this affection of his eyes. Upon inquiring into his complaints, Sir E. found them to correspond exactly with those of the former case, and therefore treated them as arising entirely from an affection of the muscles. He bound up one eye, and left the other open. The patient now saw objects single, and very distinctly, but looking at them gave him pain in the eye, and brought on headach. This led Sir E. to believe that he had erroneously tied up the sound eye: the bandage was therefore removed to the other, and that which had been bound up was left open. He now saw objects without pain or the smallest uneasiness. He was thus kept with one eye confined for a week, after which the bandage was laid aside: the disease proved to be entirely gone, nor did it return, in the smallest degree, while he remained in the hospital. Rest alone had been sufficient to allow the muscles to recover their strength, and thus to produce a cure.

"Sir Everard concludes by observing, that, when muscles are strained or over-fatigued, to put them in an easy state, and confine them from motion, is the first object of attention; and that this practice is no less applicable to the muscles of the eye, than to those of other parts."*

A species of amaurosis is mentioned, with which the profession in general may not be acquainted, namely, amaurosis from irritation of the branches of the fifth pair of nerves.

"This appears to be by no means an unfrequent cause of sympathetic amaurosis; numerous instances being on record, in which the removal of tumors in contact with branches of the fifth pair, and of carious teeth, has been the means of suddenly restoring sight.

"Case I. The daughter of a person belonging to the establishment of the Marquis of Buckingham, at Stow, about twelve years of age, was brought to Mr. Ware, on account of total blindness of the left eye, which had continued for six months without any visible cause to produce it.

"Upon the removal of a small encysted wart, which was situated on the edge of the lower eyelid, very near the punctum lachrymale, the child surprised Mr. W. by immediately saying that she had recovered her sight, and by telling him the name of every thing that was held up before her.†

"Case II. A healthy middle-aged man, a ship-painter by trade, desired Mr. Howship's advice, in 1808, on account of a small tumor situated on the crown of the head. It was at least ten years since he had first perceived it. He supposed it might have been the consequence of some blow on the part, as those in his line of business were very subject to such accidents. It had never been

* Philosophical Transactions for 1797, part I. p. 7.

† Observations on the Cataract and Gutta Serena, p. 442. London, 1812.

painful, but yet he thought his general health was giving way, as for some years he had been subject to headach; a complaint he never was afflicted with in his life before. The frequency of the headach was increasing, and his sight had become so weak, that for more than two years he had been totally unable to read even the largest and clearest print. On pressure, no pain, or even sense of feeling, was excited in the tumor on the scalp.

"Having very frequently removed such tumors, Mr. Howship advised extirpation, which was done accordingly, by carrying two elliptical incisions through the teguments beyond the basis of the tumor, the portion of included scalp, with the tumor itself, being subsequently dissected away from the pericranium, with which it was in contact. Two small vessels were tied, and the integuments brought nearly together with adhesive plaster. In three weeks the ligatures were off, and the wound perfectly healed.

"On examination, the tumor proved to be a strong cartilaginous cyst, seated in the cellular membrane beneath the scalp. The cavity of the cyst was filled with a yellow purulent fluid; the thick parts of which had formed a curdly deposit upon the sides of the cavity.

"The patient had not lost above an ounce of blood in the operation, but he rather unexpectedly felt his head better the following evening, than for many months before. He found his uneasiness and pain in the head continue to diminish from day to day, and stated, with some degree of surprise, that he also found his sight becoming much stronger, and clearer than before. By the time the wound was healed, he had quite lost all remains of pain in his head, and his sight was so greatly improved, that he was now again able to read the same small-printed book that he had been in the habit of using ten years before; nor did the pains in the head, or the affection of the sight, afterwards return.*

"Case III. F. Przesmycki, aged thirty, who had always enjoyed good health, with the exception of occasional rheumatic pains in the head and joints, was suddenly seized, in the autumn of 1825, with violent pain shooting from the left temple to the eye and side of the face. This pain was attributed to cold; it lasted several days, then subsided, but returned periodically without being so severe as to lead him to consult a medical man. But in two months it recurred with such intensity, especially in the eye, that that organ appeared to the patient about to start from its socket, and at the same time he became sensible of having lost the power of vision on that side. This discovery induced him to have recourse to professional assistance, and for six months various plans of treatment were adopted, without any other advantage than that the pain became periodical instead of continual. At the expiration of this period, the pain acquired new force, the cheek became

* Howship's Practical Observations on Surgery and Morbid Anatomy, p. 1. London, 1816.

swollen, and during the night several spoonfuls of bloody pus were discharged from between the conjunctiva and the left lower eyelid: after which the swelling subsided, and the pain diminished, but the blindness remained as complete as before. In three weeks a similar discharge took place, and during the next six months it was occasionally repeated. In the winter of 1826, the disease was so severe, that, at the commencement of 1827, the patient proceeded to Wilna, with the intention of having the eye removed, if he should find no other means of relief.

" M. Galenzowski, who was now consulted, found the vision of the left eye lost, the pupil remaining dilated. He conceived that pus had formed in the maxillary sinus, and made its way along the orbital part of the superior maxillary bone; but knowing also that suppurations of the upper jaw frequently depend upon carious teeth, a careful examination was made, and a rotten tooth found corresponding to the antrum. This tooth was extracted, to give a new outlet to the purulent matter, and, to the astonishment of M. Galenzowski and his patient, there was found attached to its root a splinter of wood, about three lines long, and as thick as the head of a pin. The splinter is supposed to have been originally detached from a toothpick of wood, as no other probable explanation could be given. The removal of a probe, introduced into the antrum, was followed by a few drops of sero-purulent fluid, and in nine days afterwards the patient completely regained his sight."* (P. 842.)

Although it is impossible we can mention all the diseases of the eye upon which Mr. Mackenzie treats, we must not terminate our notice of a work which displays such vast industry, and so much knowledge of the subject, without conveying to our readers the subjects of the various chapters. Chap. 1. Diseases of the Orbit. 2. Diseases of the secreting Lacrymal Organs. 3. Diseases of the Eyebrow and Eyelids. 4. Diseases of the Tunica Conjunctiva. 5. Diseases of the Semilunar Membrane and Caruncula Lacrymalis. 6. Diseases of the excreting Lacrymal Organs. 7. Diseases of the Muscles of the Eyeball. 8. Diseases in the Orbital Cellular Membrane. 9. Injuries of the Eyeball. 10. Inflammatory Diseases of the Eye. 11. Diseases consequent to the former. 12. Adaptation of an Artificial Eye. 13. Partial and general Enlargements of the Eyeball; Effusions and Tumors within its Coats. 14. Cataract. 15. Artificial Pupil. 16. Preternatural States of the Iris, independent of Inflammation. 17. Glaucoma and Catseye. 18. Various States of defective Vision. 19. Amaurosis.

In extensive works, we have too frequently to lament the want of an alphabetical index, without which, the labour of

* Archives Générales de Médecine, tome xxiii. p. 261. Paris, 1830.

an author is in a great measure lost; for, with a mere table of contents, it is always difficult to refer to any particular subject. Mr. Mackenzie's work is rendered additionally valuable and useful by a copious and correct index.

### COLLECTANEA.

